# Controlled roll rotation of a microparticle in a hydro-thermophoretic trap

**DOI:** 10.1103/PhysRevResearch.5.033005

**Published:** 2023-07-05

**Authors:** Gokul Nalupurackal, Kingshuk Panja, Snigdhadev Chakraborty, Srestha Roy, Jayesh Goswami, Basudev Roy, Rajesh Singh

**Affiliations:** 1 Department of Physics, Quantum Centre of Excellence for Diamond and Emergent Materials (QuCenDiEM), IIT Madras, Chennai 600036, India; 2 Department of Physics, IIT Madras, Chennai 600036, India

## Abstract

In recent years, there has been a growing interest in controlling the motion of microparticles inside and outside a focused laser beam. A hydro-thermophoretic trap was recently reported [Nalupurackal *et al*., Soft Matter **18**, 6825 (2022)], which can trap and manipulate microparticles and living cells outside a laser beam. Briefly, a hydro-thermophoretic trap works by the competition between thermoplasmonic flows due to laser heating of a substrate and thermophoresis away from the hotspot of the laser. Here, we extend that work to demonstrate the controlled roll rotation of a microparticle in a hydro-thermophoretic trap using experiments and theory. We experimentally measure the roll angular velocity of the trapped particle. We predict this *roll* rotation from theoretical computation of the fluid flow. The expression for the angular velocity fits the experimental data. Our method has potential applications in microrheology by employing a different mode of rotation.

## I Introduction

The control and manipulation of micro-/nano-objects is a key element in the field of nanophotonics [1,2], material science [3,4], biophysics [5,6], and even sensing [7]. There are several strategies that have been developed for the precise positioning and detection of such confined particle systems through optical [8–10], magnetic [11,12], acoustic [13], and thermal [14–16] means. In addition, there has been growing interest in the development of strategies to control the dynamics of microparticles in fluid-flow dominated scenarios [17–20]. In the past, people have demonstrated the indirect trapping of micro-objects by the controlled generation of pressure-driven fluidic environments or by employing potential energy landscapes and force fields generated by the temperature distributions in the particle-fluid neighborhood [21–23]. One such promising approach involves the use of hydro-thermophoretic traps [24], which can be used to trap and manipulate microparticles using a combination of fluid flow and temperature gradients.

Apart from having control over the translational degrees of freedom of a trapped particle, research groups across the globe have looked extensively into its different rotational degrees as well [25–28]. The rotational degrees are generally defined as *yaw* [29] (in-plane), *pitch* [30], and *roll* [31,32] (out-of-plane) in the nomenclature of airlines. The precise control over each rotational degree has potential applications towards multimode-microrheology [33,34], cellular tomography [35], and most importantly the assessment of surface or interface attributes in nanoscale [36–38]. Many techniques have been reported in line with the generation of rotations and torques using vortex beams [39,40], holographic optical tweezers [35], and by controlling the ellipticity of polarization [25]. These techniques have thoroughly explored the in-plane *yaw* rotation, and there are approaches that involve the generation and detection of *pitch* rotation as well [32,36,41]. For an anisotropic particle, one can distinguish between *pitch* and *roll* since Stroke’s drag is different for each mode, in proximity to a surface. Hence, it is crucial to have precise control over both of these out-of-plane rotations independently. However, out of the three rotational modes, the *roll* mode remains almost unexplored due to the difficulty in achieving it using single-beam optical tweezers. Recently, Lokesh *et al*. rotated an optically trapped particle in partial *roll* sense by switching between two different optical trap configurations [31]. Despite this, complete control of *roll* rotation has remained a challenge in a static configuration. To the best of our knowledge, controlled and continuous *roll* rotation of a microparticle outside an optical trap has not been reported yet.

**Fig. 1:**
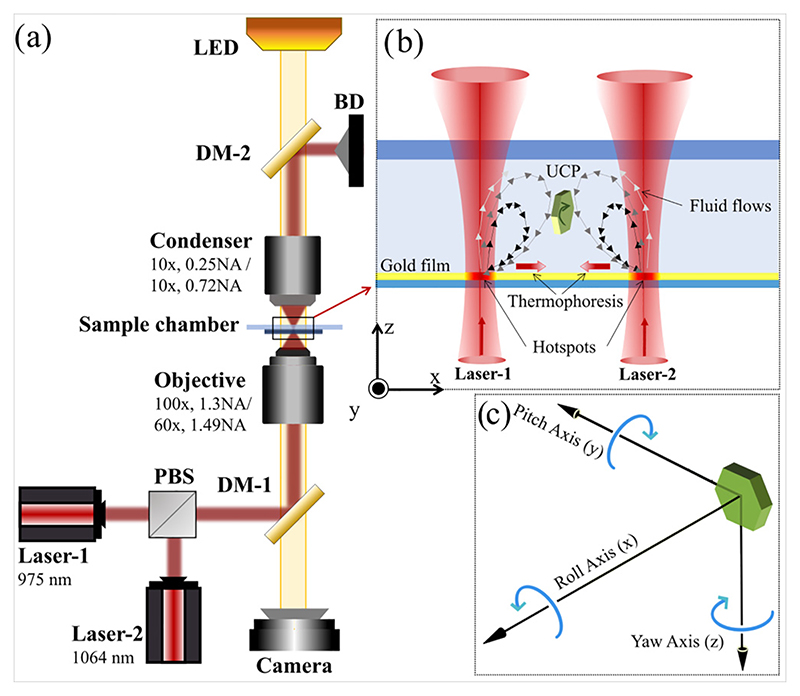
(a) The schematic diagram of the experimental setup is shown. Inset (b) shows an enlarged depiction of the sample chamber where the UCP is confined in a side-on configuration while executing out-of-plane *roll* rotation. DM denotes dichroic mirror, PBS denotes polarizing beam splitter, and BD denotes beam dump. In (c) different rotation modes of a UCP, confined in a hydro-thermophoretic trap, are shown.

In this study, we demonstrate the controlled, continuous rotation of a microparticle in *roll* sense, while being confined in a hydro-thermophoretic trap [24]. It is well known that heating a gold substrate can initiate the thermoplasmonic fluid flows, and also the temperature gradient on the same substrate can cause thermophoretic diffusion of the particle simultaneously [23,24,42]. It has been shown that a stable point is generated at the center of the line joining two laser spots (referred to as hotspots), and it can effectively confine microparticles in a quasi-three-dimensional manner. We extend this work by enhancing the thermophoretic force field away from the hotspots, thereby changing the position of the trap center peripherally, but in-plane. We observe that in this configuration, the particle tends to align along its side-on sense (see Fig. 1) and executes continuous *roll* rotations. By modulating the temperature gradient and fluid flow in the trap, we are able to precisely control this rotational motion of the particle. The angular velocity values of such dynamics are obtained at different experimental conditions through video microscopic analysis. We approach the problem both experimentally and analytically, and we derive an exact theoretical expression of fluid velocity that is shown to match the experiments. In what follows, we present our experimental system and describe our experimental and theoretical results in detail.

## II Experimental System

### A Optical tweezers setup

The experiments are carried out in inverted microscopes integrated with optical tweezers setups, namely OTKB/M from Thorlabs and Ti2 Eclipse from Nikon. The schematic of the setup is shown in Fig. 1(a).OTKB/Muses a 100×, 1.3NAoil immersion objective from Olympus for trapping, and a 10×, 0.25 NA condenser lens from Nikon for the illumination of the sample chamber, whereas the objective and condenser lenses in Nikon Ti2 eclipse are of 60×, 1.49 NA and 10×, 0.75 NA, respectively. We use lasers of wavelengths 1064 nm (Lasever, China) and 975 nm (Thorlabs, USA) to irradiate the sample chamber. Both of the lasers are directed to the sample chamber using a dichroic mirror (DM-1) and are tightly focused using the objective lens. The sample chamber is illuminated with an LED from the top through a dichroic mirror (DM-2) and imaged using a CMOS camera (Thorlabs/Nikon). The laser powers are measured at the sample stage using a power meter (PM 100D, Thorlabs).

### B Preparation of upconverting particles

The hydrothermal method is exploited to synthesize 5-μm-sized hexagonal upconverting particles (UCPs) or NaYF_4_:Er^3+^, Yb^3+^ particles [43,44]. Initially, in 14 mL of deionized water, 1.26 g of yttrium nitrate [Y(NO_3_)_3_, 63 at. % of Y] and 1.23 g of sodium citrate (Na_3_C_6_H_5_O_7_) are dissolved and stirred on a hotplate for 10 min. The aforementioned solution is then mixed with 0.38 g of ytterbium nitrate [Yb(NO_3_)_3_, 20 at. % of Yb] and 0.037 g of erbium nitrate [Er(NO_3_)_3_, 2 at. % of Er] that has been dissolved in 21 mL of the aqueous solution, resulting in a white solution. It is then converted into a clear solution by the addition of 1.411 g of sodium fluoride (NaF) in 67 mL deionized (DI) water. Magnetic stirring is kept alive for 1 h. The final solution is then transferred into a Teflon-lined autoclave and sealed tightly. The autoclave reactor is kept in a muffle furnace and heated at 200 °C for 12 h. The resulting white powder is washed with ethanol and water five times and dried at 100° to obtain the sample in pure form. We have reported the size and hexagonal geometry of the particles in our earlier works [24].

### C The experiment

The sample of upconverting particles for the experiment is prepared by dispersing them in water, 20 μL of which is transferred to a glass slide (Blue star, number 1 size, English glass) and mounted with a gold-coated coverslip (170 μm English glass + 30 nm gold) to constitute the sample chamber. It is then transferred to the sample stage of the optical tweezers setup such that the gold coating on the coverslip comes in direct contact with the sample, as shown in Fig. 1. The sample chamber is then irradiated with laser-1 and laser-2 at equal powers from the bottom. The area of the gold coating on which the laser falls gets locally heated due to the plasmonic properties of gold. In this configuration, such points of irradiation are referred to as hotspots (H1 and H2). The localized surface plasmonic heating on the gold substrate significantly increases the temperature of its surroundings, which in turn induces the convection flows in water where particles are dispersed [a schematic is shown in Fig. 1(b)]. Different modes of rotation of a confined UCP in our experiments are depicted in Fig. 1(c).

## III Results And Discussion

In this section, we describe our main experimental and theoretical results.

### A Controlled roll rotation of a microparticle

**Fig. 2:**
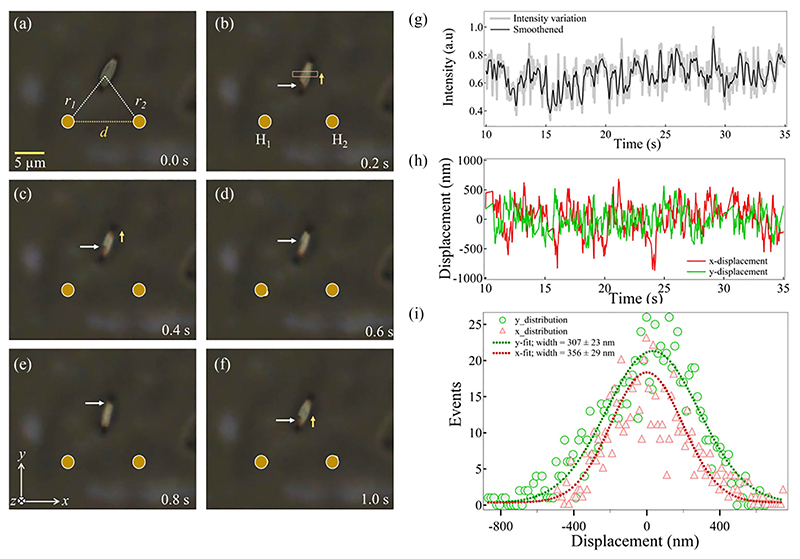
Parts (a)–(f) show the *roll* rotation of a 5-micron-sized UCP in fluid flow, spatially confined at a vertical point from the line joining two hotspots H1 and H2. A small dent on the particle pointed out with a white arrowhead can be taken as a reference to visualize such rotation. The total intensity variation on the particle across a rectangular box is plotted as a function of time in (g). In (h) and (i), the *x,y*-displacement time series and the corresponding positional distributions, fitted with a Gaussian, are shown. The trap stiffnesses along the *x* and *y* directions are extracted and are found to be *k_x_* = 32.6 ± 5.3 fN/μm and *k_y_* = 43.9 ± 6.4 fN/μm. The laser power at each hotspot is measured to be 5 mW. The above dynamics are shown in the Supplemental movie I [45].

In Figs.2(a)–2(f), we experimentally show the controlled out-of-plane rotation of a single upconverting particle in *roll* sense confined in a hydro-thermophoretic trap, repositioned just above the line joining two hotspots (H1 and H2) in the *x*-*y* plane. The laser powers at each hotspot (P1 and P2) are fixed at 5 mW, and the separation between them (*d*) is changed [thus to change *l* and sin *θ* in Eq. (13)].

**Fig. 3:**
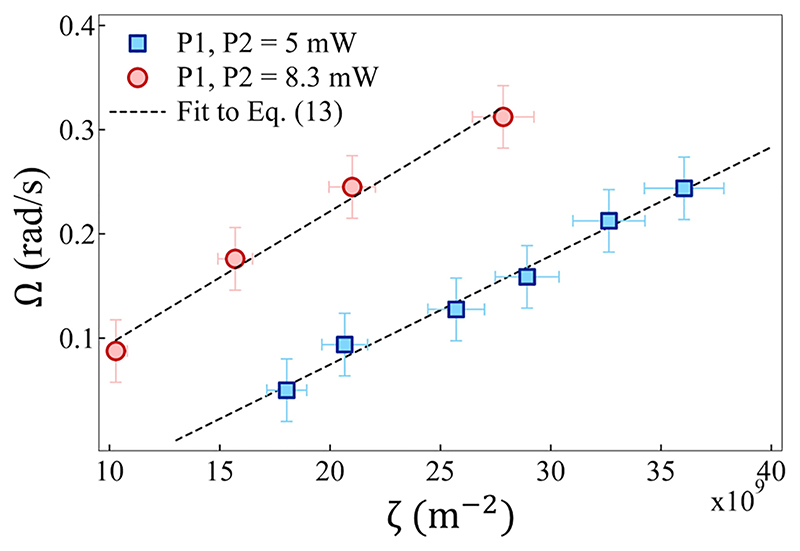
The magnitude of the angular velocity Ω of the confined particle at two different laser powers is plotted as a function of ζ. Note that P1 = P2. The value of *F*_0_ is calculated to be 33.8 ±5.3 fN (at 5 mW) and 58.2 ±7.1 fN (at 8.3 mW) by fitting the experimental values to Eq. (13) at the two values of the laser power.

The particle is confined in a side-on configuration in such a way that the rotation is observed about the *x*-axis of the experimental system [defined as *roll* rotation in our experiments, Fig. 1(c)]. The average angular velocity (Ω) of such rotation is determined by tracking the position of one of the edges of the particle using MATLAB and IMAGEJ software and dividing it by half the diagonal length (2.5 μm). To visualize the rotation more precisely, we also trace the total intensity of a rectangular patch marked on the particle [see Fig. 2(b)] and it is shown in Fig. 2(g). The in-plane displacement time series of the particle (*x* and *y*) are generated by tracking its centroid as shown in Fig. 2(h). We also determine *x* and *y* positional distributions of the particle to confirm its confinement and fit them with Gaussian curves [Fig. 2(i)]. The trap stiffness values are calculated from the fits and are found to be *k_x_* = 32.6 ±5.3 fN/μm and *k_y_* = 43.9 ±6.4 fN/μm. By taking account of the observation that the particle’s displacement within the trap is on the orders of micrometers, we are simultaneously applying and estimating the hydro-thermophoretic forces on the order of femto-newton here. Further, the average angular velocity values are determined by changing *d* and plotted as a function of a scaling variable *ζ* in Fig. 3 (see Sec. III C for details).

To ascertain the laser power dependence of hydro-thermophoretic forces and the angular velocity of the particle, we perform the same experiment with elevated powers at the hotspots. The new power readings at H1 and H2 are made to be 8.5 mW. Again, the estimated angular velocity values are plotted as a function of ζ and fitted with Eq. (13) as shown in Fig. 3. The slope of such a linear fit yields the magnitude of average force at the trap position (*F*_0_) due to thermoplasmonic fluid flows. We observe that the force of confinement increases with an increase in laser power at both hotspots, not to mention the increase in angular velocity. These effects need to be studied qualitatively and will be pursued as a separate work in the future.

### B Tuning the location of the hydro-thermophoretic trap

**Fig. 4:**
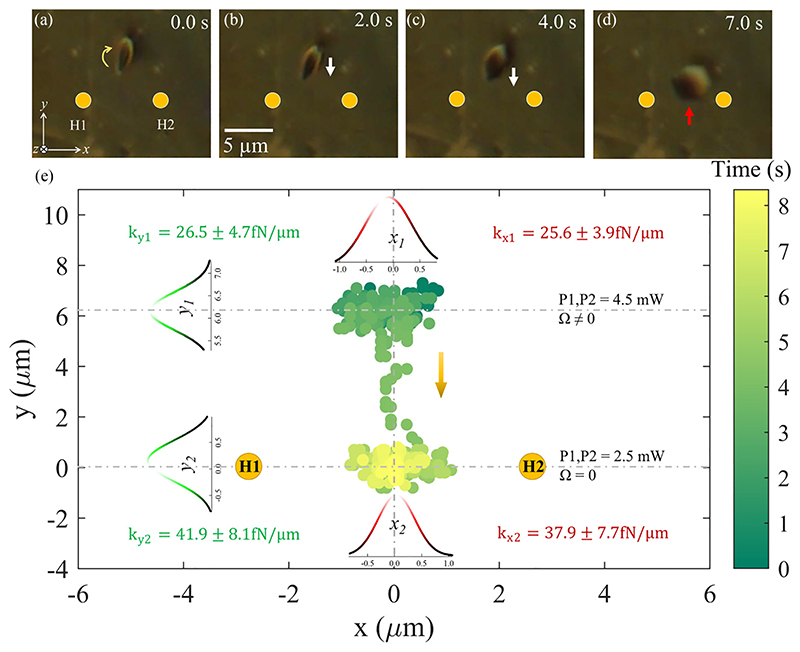
Parts (a)–(d) show the transition of the particle between two equilibrium positions. In (a), the laser power at each hotspot is kept at 4.5 mW (Ω = 0). As the power is slowly brought down to 2.5 mW, the particle reaches another equilibrium position between the line joining two hotspots. The average angular velocity along the *x*-direction is observed to be zero at this position. In (e), the *x*-*y* in-plane displacement of the particle as a function of time is shown. The dynamics corresponding to this figure can also be seen in Supplemental movie II [45].

Here, we show that the location of the hydro-thermophoretic trap can be tuned exclusively by changing the laser power at both hotspots. Initially, the laser powers at each hotspot (H1 and H2) are kept at 4.5 mW. We observe that the particle is getting confined above the line joining hotspots (*l* = 6.2 μm). The continuous *roll* rotational motion of the particle about the *x*-axis is also observed as shown in Fig. 4(a), and Ω of such rotations are estimated.

As we decrease the laser power at each hotspot to 2.5 mW, the particle traces a downward trajectory (along the –*y* axis) and gets confined exactly to the middle of the line joining two hotspots, bringing *l* = 0, sin *θ* = 0 (see Sec.III C 2 for theoretical descriptions). This happens due to a competition between the thermophoretic velocity and advection by the thermoplasmonic flow, resulting in a unique stable point that relies on the heat generated at the hotspots. In this manner, the position of the hydro-thermophoretic trap can be controlled vertically [see Figs.4(b)-4(d)]. The average angular velocity of the particle ceases as it repositions in an aforementioned manner. It is also evident from Eq. (13) that the angular velocity vanishes as the particle gets closer to the exact middle of the line (*l* = 0).

The *x* and *y* displacements of the particle are tracked as a function of time throughout the course of the experiment, the distributions of which at the stable positions yield the trap stiffness values ([*k*_*x*1_ *k*_*y*1_] and [*k*_*x*2_, *k*_*y*2]_) that are shown in Fig. 4(e). The forces are determined to be on the order of femto-newtons here as well.

### C Mechanism of flow-induced roll rotation

In this section, we obtain an analytical expression of the angular velocity of the trapped microparticles by studying the equations of motion.

#### 1 Governing equations for fluid and heat flows

**Fig. 5:**
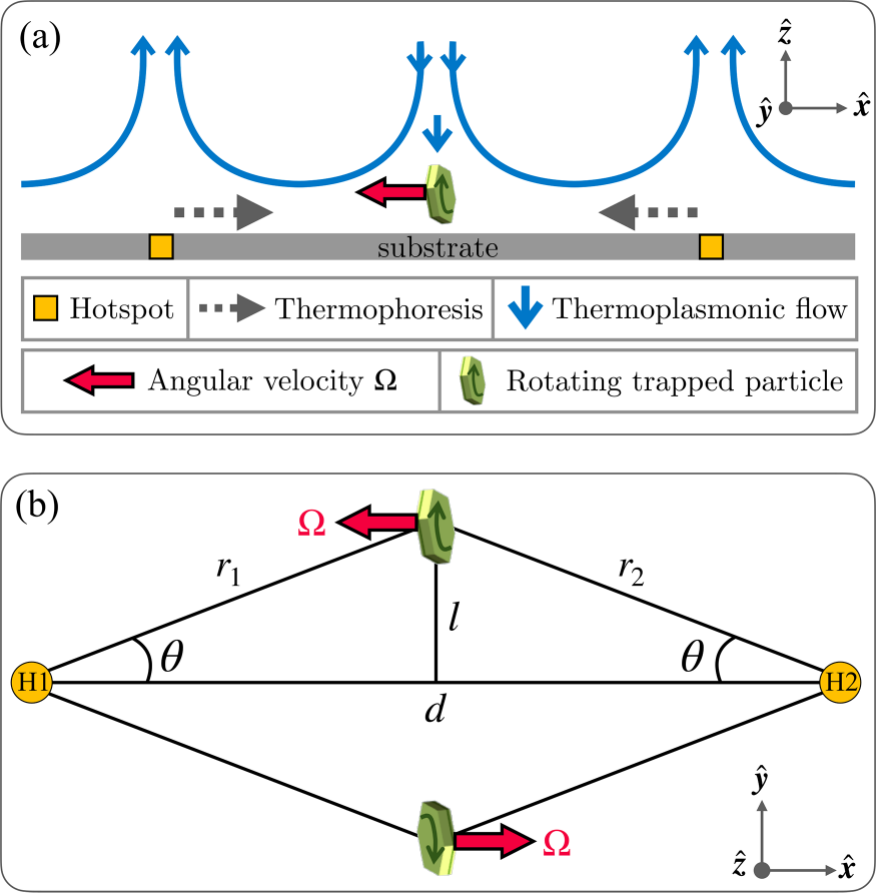
(a) Schematic diagram for the mechanism of rotation in a roll-sense for a trapped UCP particle through the combined effects of thermophoresis and thermoplasmonic flows. Hotspots 1 and 2 are marked by H1 and H2, respectively. The two hotspots are separated by a distance *d* as shown in (b). Panel (b) also shows schematically that there is a net angular velocity **Ω** for *l* = 0 or equivalently *θ* = 0 [see Eq. (13) and related text for details].

We now study the fluid flows produced by plasmonic heating of the gold substrate through laser radiations (see Fig. 5 for a schematic diagram of the system). The fluid flow ***v***(***s***) at a point ***s*** in the bulk fluid satisfies the Stokes equation [46] in the limit of low Reynolds number (as applicable to our experimental system):

(1) $$-\nabla p+\eta \nabla^{2}v=-f,$$

where *p* is the fluid pressure, *η* is the viscosity, and $f=\rho \beta g\delta T\hat{z}$ is the force density in the fluid due to the plasmonic heating of the gold substrate, which is obtained from the Boussinesq approximation for buoyancy-driven natural convection [47–49]. Here *ρ* is the fluid density, *β* is the thermal expansion coefficient, and *g* is the acceleration along with δ*T* = (*T* – *T*_0_). The fluid is incompressible, **V** · *v* = 0. In addition, the fluid flow vanishes at the plane walls of the experimental chamber. There is a reduction of mass density around the gold-coated substrate because of heating by the laser, which yields the upward convection of the fluid driven by the force density ***f***, defined above. The fluid flow is driven by the plasmonic heating of the gold substrate through laser radiations of source densities *q_i_* [49–52]. The temperature field in the steady state, at any point on the substrate, is given by the solution of the following equation [50,51]:

(2) $$\nabla \cdot (\kappa \nabla T)=-\left(q_{1}+q_{2}\right).$$

Here *κ* is the thermal conductivity, while *q*_1_ and *q*_2_ are the heat source density which serves as a source term for the computation of the temperature field *T*(***s***). The total heat power generated on the hotspot from the two lasers is *Q*_1_ and *Q*_2_, where *Q_i_* = *∫* *q*(***s***) *d**s***.

The laser heating of the gold-coated substrate results in a temperature gradient that is maintained even outside the laser beam because a gold-coated substrate has high thermal conductivity. Indeed, all our experiments are performed on a gold-coated substrate for this very reason. Thus, a microparticle in the vicinity of the substrate executes a thermophoretic motion [53,54] even if it is not directly inside the laser beam. The thermophoretic velocity of a microparticle is given as [53–56],

(3) $$v_{\text{T}}=-D_{\text{T}}\nabla T,$$

where *D*_T_ is the thermophoretic mobility [53]. For our experimental system, the thermophoretic velocity *v*_T_ is away from the hotspot. In [24], it was shown that the combined effects of thermoplasmonic flows and thermophoresis act as a hydro-thermophoretic trap for a microparticle. See Fig. 5(a) for a schematic describing the mechanism of the hydro-thermophoretic trap. The thermoplasmonic flows push the particle towards the hotspot, while near the hotspot thermophoresis dominates, pushing the particle away from the hotspot. The balance of these two competing effects leads to the trapping of the particle. The schematic also contains the direction of the angular velocity of the trapped particle. In what follows, we compute the angular velocity of a trapped particle. We show that the direction and magnitude of the angular velocity **Ω** of the trapped particle can be controlled by tuning the location of the trap center.

#### 2 Angular velocity of the trapped microparticle

The fluid flow *v* is induced by thermoplasmonic heating of the gold-substrate, as per the Stokes equation given in Eq. (1). We model the resulting force density in the fluid as point forces. Thus, the force density ***f*** at a point ***s*** in the fluid is given as

(4) $$f(s)=F_{0}[\delta (s-R_{1})+\delta (s-R_{2})]\hat{z}.$$

Here *F*_0_ is a constant strength of the point forces located at ***R**_i_*, where *i* = 1, 2. The location ***R**_i_* of the *i*th point force is chosen to be at the center of the *i*th hotspot, while both point forces are located at a height *h* from the substrate. This modeling of thermoplasmonic flows by a point force (or a Stokeslet) was first done in [57]. We note that the strength *F*_0_ is expected to increase with the increasing power of the laser. The fluid velocity at a field point ***s*** can then be written as a sum of contributions from two-point forces at point ***R***_1_ and ***R***_2_ [58]. The explicit form of the fluid velocity *v* at a point ***r*** is then given as

(5) $$v(s)=G(s,R_{1})\cdot F_{1}+G(s,R_{2})\cdot F_{2}.$$

Here $F_{1}=F_{2}=F_{0}\hat{z}$, while ***G*** is a Green’s function of Stokes flow, which is defined as [58]

(6) $$\nabla_{\beta }G_{\alpha \beta }(s,s^{\prime })=0,$$

(7) $$\nabla_{\alpha }P_{\beta }(s,s^{\prime })+\eta \nabla^{2}G_{\alpha \beta }(s,s^{\prime })=\delta (s-s^{\prime })\delta_{\alpha \beta }$$

Thus, we need to choose a Green’s function of Stokes equations, which ensures that the fluid is incompressible **Δ** · *v* = **0**. In addition, it should ensure the no-slip boundary condition or the fact that the fluid flow must vanish at the substrate. Thus, we must have *v* = **0** at the substrate, which is chosen to be at *z* = 0. A Green’s function of the Stokes equation, which satisfies these properties, for a source at a height *h* from the wall, can be written as

(8) $$G_{\alpha \beta }(R,R^{\prime })=G_{\alpha \beta }^{\text{o}}(r)+G_{\alpha \beta }^{*}(R,R^{*}).$$

Here r* = ***R*** – R*, R* = 𝓜 · ***R***′ is the image at a distance *h* from the wall at *z* = 0. Note, $ℳ=I-2\hat{z}\hat{z}$,

(9) $$G_{\alpha \beta }^{o}(r)=\frac{1}{8\pi \eta }\left(\frac{\delta_{\alpha \beta }}{r}+\frac{r_{\alpha }r_{\beta }}{r^{3}}\right)$$

is the Oseen tensor [59,60] with *r* = |***r***| = |***R*** – ***R***′|, and G* is the correction necessary to satisfy the boundary condition [61]. The explicit form is

(10) $$G_{\alpha \beta }^{*}(R,R^{*})=-G_{\alpha \beta }^{\text{o}}(r^{*})-2h\nabla_{r_{\gamma }}G_{\alpha 3}^{\text{o}}(r^{*}){ℳ}_{\beta \gamma }+h^{2}\nabla_{r^{*}}^{2}G_{\alpha \gamma }^{\text{o}}(r^{*}){ℳ}_{\beta \gamma }.$$

Using the above expression of the Green’s function in Eq. (5), we obtain the net fluid flow at any point in the system. We emphasize that this flow vanishes identically at the substrate.

The angular velocity **Ω** of the particle follows from the vorticity ***ω*** in the fluid at the location of the particle. It is given as

(11) $$\text{Ω}=\frac{1}{2}\omega ,\;\omega =\nabla \times v.$$

Here we use the fact that a steady-state temperature field cannot induce any rotation of the particle. Thus, we only need to focus on the fluid flow to obtain the angular velocity of the particle. The vorticity of the fluid can be obtained by using Eqs. (5), (10), and (11) once the forces at the two hotspots—modeled as point forces in the fluid—are given. The vorticity in the fluid is

(12) $$\omega =-\frac{F_{0}}{4\pi \eta }\left[\left(\frac{1}{r_{1}^{3}}-\frac{1}{r_{1}^{*3}}\right)(r_{1}\times \hat{z})\right]+\frac{F_{0}}{4\pi \eta }\left[\left(\frac{1}{r_{2}^{3}}-\frac{1}{r_{2}^{*3}}\right)(r_{2}\times \hat{z})\right].$$

**Fig. 6:**
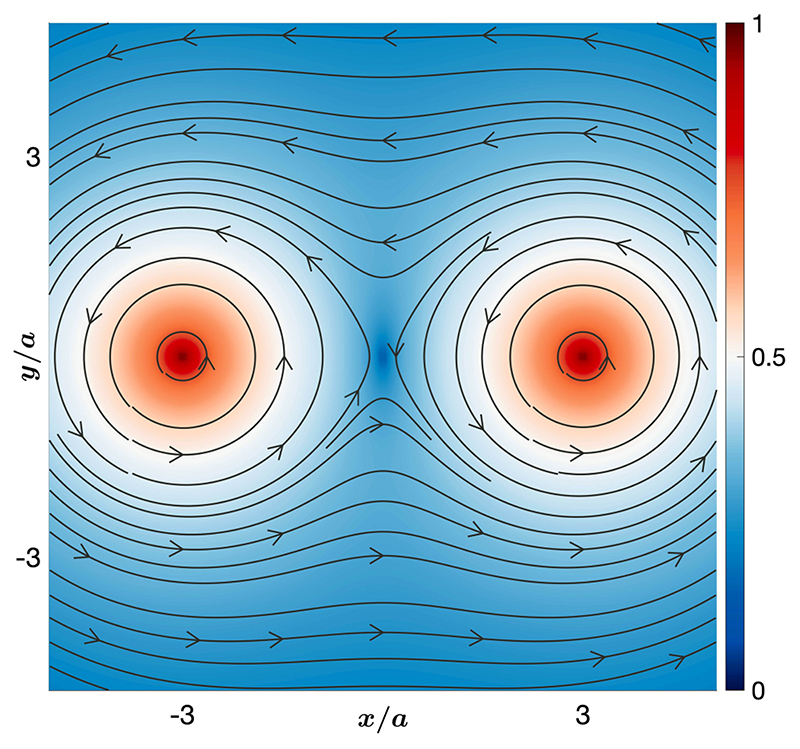
Vector field plot of the vorticity **ω** plotted in a plane parallel at height *h* =1 .4*a* from the substrate. The flow is driven by the two hotspots at *x* = ±3*a*. The vorticity vector field is overlaid on a pseudocolor plot of the magnitude of the vorticity, which is normalized to the maximum. The center point of the line joining the hotspots is a point of zero vorticity. On the other hand, field points above and below this line are directions of opposite vorticity.

Here ***r**_i_* is the distance of the field point from the *i*th hotspot, where *i* = 1, 2, while $r^{*}=\sqrt{r^{2}+4h^{2}}$ along with the fact that *h* is the height of the particle and point force from the substrate. We use *h =* 1.4*a*, where *a* is the radius (diagonal length for a UCP) of the particle. The above expression for the vorticity is plotted in Fig. 6. It is clear that the vorticity vanishes at *x* = *y* = 0, which is also the center of the line joining the hotspots. Moreover, for the plane *x* = 0, the vorticity vector points along the negative $\hat{x}$-direction for *y* > 0 and the positive $\hat{x}$-direction for *y* < 0. The vorticity vanishes identically at the point *x* = *y* = 0. Thus, we have qualitatively explained the experimental rotational dynamics.

To obtain a quantitative match with the experimental data, we obtain an explicit form of the angular velocity of a particle trapped in the hydro-thermophoretic trap. We first note that the distance of the trapped particle is the same from the two hotspots. This is because, throughout this work, we keep the power at the two hotspots the same. Thus by symmetry *r*_1_ = *r*_2_ = *r* and $r_{1,x}=-r_{2,x}=\frac{d}{2}$. Using the above expression of the vorticity, we obtain the angular velocity of the trapped particle as

(13) $$\text{Ω}=-\frac{F_{0}}{4\pi \eta }\left(\frac{1}{r^{3}}-\frac{1}{r^{*3}}\right)l\hat{x}=-\frac{F_{0}}{4\pi \eta }\zeta \hat{x},$$

where we have defined

$$\zeta =\left(\frac{1}{r^{2}}-\frac{r}{r^{*3}}\right)\sin\theta .$$

In the above, we have also used the relation *l* = *r* sin *θ*; see Fig. 5(b) for a schematic. The experimentally measured angular velocity matches very well with the theoretical predictions in Eq. (13). This is shown in Fig. 3. We note that a particle of extended shape will perform a rotation in roll sense in this configuration. Moreover, the speed of the rotation depends on the power of the laser through the constant *F*0 in Eq. (13), which is proportional to the laser power. Thus, the slope of a plot of angular speed with ζ (defined in the above equation) will be higher at a higher value of power. This is in agreement with the observations in the experiment; see Fig. 3. It is clear from the above expression [Eq. (13)] that the angular velocity vanishes when the particle is trapped along the line joining the hotspots such that sin *θ* = 0. On the other hand, the angular velocity is nonzero when the particle is pushed above or below the line joining the hotspots by increasing the laser power. From the expression Eq. (13) and Fig. 6, the microparticle rotates with angular velocity **Ω** pointing along the negative $\hat{x}$-axis for *y* > 0 and with angular velocity **Ω** pointing along the positive $\hat{x}$-axis for *y* < 0. See Fig. 5(b) for a schematic representation of this result. This theoretical result is in excellent agreement with experimental observations that the sense of rotation changes if the particle is trapped below or above the line joining the hotspots. Thus, the *roll* rotation of the trapped microparticle observed in the experiments is captured by our model of the thermoplasmonic fluid flow, both qualitatively (see Fig. 6) and quantitatively (see Fig. 3).

## IV Summary And Outlook

To summarize, we have presented an experimental method to generate a controlled and continuous out-of-plane rotation of a microparticle in the *roll* sense. This microparticle (a hexagonal UCP) performs *roll* rotations while confined in a quasi-3D hydro-thermophoretic trap. The *roll* rotational motion of the microparticle is due to the convection currents of thermoplasmonic fluid flows. We show that a simple model for thermoplasmonic fluid flows can predict the angular velocity of the microparticle. In our model, the fluid flow driven by the laser heating of each hotspot can be captured by a point force in the fluid near the substrate. The predicted angular velocity from this model is in excellent agreement with the experimental observations. Thus, using a combined theoretical and empirical analysis, we have demonstrated that the roll rotations of a particle trapped in a hydro-thermophoretic trap can be tuned to desirable values.

Our results show that this approach could be used to develop new techniques for the manipulation and control of microparticles in fluidic environments. This could have important applications in fields such as microfluidics, biophysics, and materials science. We believe that this technique could have important implications for the development of new tools and techniques for the manipulation and control of microparticles in fluidic environments. Moreover, the controlled rotation of microparticles may be used to study nonequilibrium statistical mechanics in colloidal systems [62]. The combined theoretical and experimental method presented here is also applicable to study the microrheology of colloidal suspensions while focusing on the distinct modes of rotational dynamics. For instance, this can be used to find surface properties [33,36]. These directions provide exciting avenues for future work.

## Supplementary Material

Movie 1

Movie 2

## References

[1] Gao D, Ding W, Nieto-Vesperinas M, Ding X, Rahman M, Zhang T, Lim C, Qiu C-W (2017). Optical manipulation from the microscale to the nanoscale: Fundamentals, advances and prospects. Light Sci Appl.

[2] Xavier J, Vincent S, Meder F, Vollmer F (2018). Advances in optoplasmonic sensors–combining optical nano/microcavities and photonic crystals with plasmonic nanostructures and nanoparticles. Nanophotonics.

[3] Lin L, Peng X, Zheng Y (2017). Reconfigurable optothermoelectric printing of colloidal particles. Chem Commun.

[4] Xie Y, Rufo J, Zhong R, Rich J, Li P, Leong KW, Huang TJ (2020). Microfluidic isolation and enrichment of nanoparticles. ACS Nano.

[5] Favre-Bulle IA, Stilgoe AB, Scott EK, Rubinsztein-Dunlop H (2019). Optical trapping in vivo: theory, practice, and applications. Nanophotonics.

[6] Choudhary D, Mossa A, Jadhav M, Cecconi C (2019). Bio-molecular applications of recent developments in optical tweezers. Biomolecules.

[7] Kayci M, Chang H-C, Radenovic A (2014). Electron spin resonance of nitrogen-vacancy defects embedded in single nanodiamonds in an abel trap. Nano Lett.

[8] Neuman KC, Block SM (2004). Optical trapping. Rev Sci Instrum.

[9] Volpe G, Maragò OM, Rubinzstein-Dunlop H, Pesce G, Stilgoe AB, Volpe G, Tkachenko G, Truong VG, Chormaic SN, Kalantarifard F (2023). Roadmap for optical tweezers. J Phys Photon.

[10] Kumar G When plasmonic colloids meet optical vortices–a brief review.

[11] Gosse C, Croquette V (2002). Magnetic tweezers: Micromanipulation and force measurement at the molecular level. Biophys J.

[12] Strick TR, Allemand J-F, Bensimon D, Bensimon A, Croquette V (1996). The elasticity of a single supercoiled dna molecule. Science.

[13] Hertz HM (1995). Standing-wave acoustic trap for nonintrusive positioning of microparticles. J Appl Phys.

[14] Fränzl M, Cichos F (2022). Hydrodynamic manipulation of nano-objects by optically induced thermo-osmotic flows. Nat Commun.

[15] Braun M, Würger A, Cichos F (2014). Trapping of single nanoobjects in dynamic temperature fields. Phys Chem Chem Phys.

[16] Paul D, Chand R, Kumar GVP (2022). Optothermal evolution of active colloidal matter in a defocused laser trap. ACS Photon.

[17] Lutz BR, Chen J, Schwartz DT (2006). Hydrodynamic tweezers: 1. Noncontact trapping of single cells using steady streaming microeddies. Anal Chem.

[18] Jiang H-R, Yoshinaga N, Sano M (2010). Active Motion of a Janus Particle by Self-Thermophoresis in a Defocused Laser Beam. Phys Rev Lett.

[19] Stoev ID, Seelbinder B, Erben E, Maghelli N, Kreysing M (2021). Highly sensitive force measurements in an optically generated, harmonic hydrodynamic trap. eLight.

[20] Shenoy A, Rao CV, Schroeder CM (2016). Stokes trap for multiplexed particle manipulation and assembly using fluidics. Proc Natl Acad Sci USA.

[21] Būtaitė UG, Gibson GM, Ho Y-LD, Taverne M, Taylor JM, Phillips DB (2019). Indirect optical trapping using light driven micro-rotors for reconfigurable hydrodynamic manipulation. Nat Commun.

[22] Tanyeri M, Johnson-Chavarria EM, Schroeder CM (2010). Hydrodynamic trap for single particles and cells. Appl Phys Lett.

[23] Luan Q, Macaraniag C, Zhou J, Papautsky I (2020). Microfluidic systems for hydrodynamic trapping of cells and clusters. Biomicrofluidics.

[24] Nalupurackal G, Gunaseelan M, Roy S, Lokesh M, Kumar S, Vaippully R, Singh R, Roy B (2022). A hydro-thermophoretic trap for microparticles near a gold-coated substrate. Soft Matter.

[25] Friese MEJ, Nieminen TA, Heckenberg NR, Rubinsztein-Dunlop H (1998). Optical alignment and spinning of laser-trapped microscopic particles. Nature (London).

[26] Ramaiya A, Roy B, Bugiel M, Schäffer E (2017). Kinesin rotates unidirectionally and generates torque while walking on microtubules. Proc Natl Acad Sci USA.

[27] Zhong M-C, Zhou J-H, Ren Y-X, Li Y-M, Wang Z-Q (2009). Rotation of birefringent particles in optical tweezers with spherical aberration. Appl Opt.

[28] Lee YV, Wu D, Fang Y, Peng Y, Tian B (2020). Tracking longitudinal rotation of silicon nanowires for biointerfaces. Nano Lett.

[29] Vaippully R, Lokesh M, Roy B (2021). Continuous rotational motion in birefringent particles using two near-orthogonally polarized optical tweezers beams at different wavelengths with low ellipticity. J Opt.

[30] Roy B, Ramaiya A, Schäffer E (2018). Determination of pitch rotation in a spherical birefringent microparticle. J Opt.

[31] Lokesh M, Nalupurackal G, Roy S, Chakraborty S, Goswami J, Gunaseelan M, Roy B (2022). Generation of partial roll rotation in a hexagonal NaYF_4_ particle by switching between different optical trapping configurations. Opt Express.

[32] Nalupurackal G, Murugan G, Lokesh M, Vaippully R, Chauhan A, Nanda BRK, Sudakar C, Kotamarthi HC, Datta P, Sinha Mahapatra P (2023). Simultaneous optical trapping and electromagnetic micromanipulation of ferromagnetically doped NaYF_4_ microparticles. ACS Appl Opt Mater.

[33] Vaippully R, Ramanujan V, Gopalakrishnan M, Bajpai S, Roy B (2020). Detection of sub-degree angular fluctuations of the local cell membrane slope using optical tweezers. Soft Matter.

[34] Roy S, Vaippully R, Lokesh M, Nalupurackal G, Edwina P, Bajpai S, Roy B (2023). Comparison of translational and rotational modes towards passive rheology of the cytoplasm of MCF-7 cells using optical tweezers. Front Phys.

[35] Lin Y-C, Chen H-C, Tu H-Y, Liu C-Y, Cheng C-J (2017). Optically driven full-angle sample rotation for tomographic imaging in digital holographic microscopy. Opt Lett.

[36] Lokesh M, Vaippully R, Nalupurackal G, Roy S, Bhallamudi VP, Prabhakar A, Roy B (2021). Estimation of rolling work of adhesion at the nanoscale with soft probing using optical tweezers. RSC Adv.

[37] Jose M, Lokesh M, Vaippully R, Satapathy DK, Roy B (2022). Temporal evolution of viscoelasticity of soft colloid laden air–water interface: A multiple mode microrheology study. RSC Adv.

[38] Schäffer E, Nørrelykke SF, Howard J (2007). Surface forces and drag coefficients of microspheres near a plane surface measured with optical tweezers. Langmuir.

[39] Gecevičius M, Drevinskas R, Beresna M, Kazansky PG (2014). Single beam optical vortex tweezers with tunable orbital angular momentum. Appl Phys Lett.

[40] Santamato E, Sasso A, Piccirillo B, Vella A (2002). Optical angular momentum transfer to transparent isotropic particles using laser beam carrying zero average angular momentum. Opt Express.

[41] Lokesh M, Vaippully R, Bhallamudi VP, Prabhakar A, Roy B (2021). Realization of pitch-rotational torque wrench in two-beam optical tweezers. J Phys Commun.

[42] Lin L, Wang M, Peng X, Lissek EN, Mao Z, Scarabelli L, Adkins E, Coskun S, Unalan HE, Korgel BA (2018). Optothermoelectric nanotweezers. Nat Photon.

[43] Kumar S, Kumar A, Gunaseelan M, Vaippully R, Chakraborty D, Senthilselvan J, Roy B (2020). Trapped in out-of-equilibrium stationary state: Hot brownian motion in optically trapped upconverting nanoparticles. Front Phys.

[44] Kumar S, Gunaseelan M, Vaippully R, Kumar A, Ajith M, Vaidya G, Dutta S, Roy B (2020). Pitch-rotational manipulation of single cells and particles using single-beam thermo-optical tweezers. Biomed Opt Exp.

[45] http://link.aps.org/supplemental/10.1103/PhysRevResearch.5.033005.

[46] Landau LD, Lifshitz EM (1959). Fluid Mechanics.

[47] Donner JS, Baffou G, McCloskey D, Quidant R (2011). Plasmon-assisted optofluidics. ACS Nano.

[48] Ndukaife JC, Kildishev AV, Nnanna AGA, Shalaev VM, Wereley ST, Boltasseva A (2016). Long-range and rapid transport of individual nano-objects by a hybrid electrothermoplasmonic nanotweezer. Nat Nanotechnol.

[49] Roxworthy BJ, Bhuiya AM, Vanka SP, Toussaint KC (2014). Understanding and controlling plasmon-induced convection. Nat Commun.

[50] Govorov AO, Richardson HH (2007). Generating heat with metal nanoparticles. Nano Today.

[51] Baffou G, Quidant R (2013). Thermo-plasmonics: Using metallic nanostructures as nano-sources of heat. Laser Photon Rev.

[52] Baffou G, Quidant R, García de Abajo FJ (2010). Nanoscale control of optical heating in complex plasmonic systems. ACS Nano.

[53] Anderson JL (1989). Colloid transport by interfacial forces. Annu Rev Fluid Mech.

[54] Würger A (2010). Thermal non-equilibrium transport in colloids. Rep Prog Phys.

[55] Braibanti M, Vigolo D, Piazza R (2008). Does Thermophoretic Mobility Depend on Particle Size?. Phys Rev Lett.

[56] Piazza R (2008). Thermophoresis: Moving particles with thermal gradients. Soft Matter.

[57] Namura K, Nakajima K, Suzuki M (2017). Quasi-stokeslet induced by thermoplasmonic marangoni effect around a water vapor microbubble. Sci Rep.

[58] Pozrikidis C (1992). Boundary Integral and Singularity Methods for Linearized Viscous Flow.

[59] Dhont JKG (1996). An Introduction to Dynamics of Colloids.

[60] Happel J, Brenner H (1965). Low Reynolds Number Hydrodynamics: With Special Applications to Particulate Media.

[61] Blake JR (1971). A note on the image system for a Stokeslet in a no-slip boundary. Math Proc Cambridge Philos Soc.

[62] Bechinger C, Di Leonardo R, Löwen H, Reichhardt C, Volpe G, Volpe G (2016). Active particles in complex and crowded environments. Rev Mod Phys.

